# Sex cord ovarian tumours over 10 years: a retrospective analysis of clinicopathological profile and outcome

**DOI:** 10.3332/ecancer.2024.1769

**Published:** 2024-09-16

**Authors:** Mona Naman Shah, Vinotha Thomas, Anjana Joel, Reka Karuppusami, Dhanya Susan Thomas, Ajit Sebastian, Anitha Thomas, Rachel Chandy, Abraham Peedicayil

**Affiliations:** 1 Department of Gynaecologic Oncology, Christian Medical College, Vellore 632004, India; 2 Department of Medical Oncology, Christian Medical College, Vellore 632004, India; 3 Department of Biostatistics, Christian Medical College, Vellore 632004, India; a https://orcid.org/0009-0005-2019-7132; b https://orcid.org/0000-0002-0858-2995; c https://orcid.org/0000-0002-6821-5900; d https://orcid.org/0000-0001-9913-2713; e https://orcid.org/0000-0003-2230-530X; f https://orcid.org/0000-0002-0145-6654; g https://orcid.org/0000-0002-5533-0184; h https://orcid.org/0000-0002-5040-8499; i https://orcid.org/0000-0002-9318-566x

**Keywords:** sex cord ovarian tumours, granulosa cell tumours, Sertoli Leydig tumours

## Abstract

**Objectives:**

To retrospectively describe the clinicopathological profile and treatment outcome of sex cord ovarian tumours (SCOTs), from a single institution.

**Methods:**

Patients who operated for SCOT between January 2011 and December 2020 were identified from the institution’s discharge summaries. Treatment details and oncologic outcomes were analyzed using descriptive statistics, SPSS statistics version 21. Progression-free survival and overall survival were plotted using the Kaplan–Meier method.

**Results:**

Over 10 years, 120 patients underwent surgery with 73 (61%) malignant SCOTs. Eight (6.6%) were referred with recurrence. Granulosa cell histology (61/73, 83.5%) and federation of gynaecology and obstetrics (FIGO) stage I disease (57/65, 78.62%) were predominant. Three (3/26,11.53%) had lymph node involvement. Adjuvant chemotherapy was advised in 53.4% (39/73).

Over a median period of 47 months (1–130 months), eleven (15.06%) patients recurred (5-year recurrence rate: 9.58%) and 6 died (5-year survival rate: 89.04%).

Among 65 patients with upfront disease, 9 (13.8%) recurred over a median period of 46 months (1–65 months) with 4 disease-related deaths. On univariate analysis, incomplete cytoreduction hazard ratios (HR 58.391, 95% CI 5.042–674.854), advanced FIGO stage (HR 15.931, 3.74–67.89) and nongranulosa histology was associated with recurrence. On multivariate analysis, advanced FIGO stage (HR 20.099, 95% CI 3.75–107.711) and non granulosa histology (HR 31.35, 95% 2.801–350.897 ) remained significant. Lymphadenectomy and adjuvant chemotherapy did not prevent recurrence.

## Conclusion

Despite a favourable 5-year survival rate, non granulosa histology and advanced-stage SCOTs pose higher recurrence risks. Personalized decision-making by gynaecologic oncologists is crucial for lymphadenectomies and adjuvant chemotherapy due to unproven benefits. The establishment of rare ovarian tumour registries is encouraged.

## Background

Sex cord ovarian tumours (SCOTs) are a rare entity among non epithelial ovarian tumours and are usually found in young adult and middle-aged women. They are characterized by endocrine manifestations at presentation along with signs and symptoms of pelvic mass. These heterogeneous tumours, consist of various pathological types and include both benign and malignant. The malignant tumours are, often non metastatic and confined to the ovary at presentation but are characterised by an indolent course and late recurrences.

Over time, with increased reporting of retrospective cohort studies, treatment decision making has evolved. As a sizable proportion of patients with SCOTs are young, fertility preservation surgery with contralateral ovarian conservation has been found to have acceptable oncological outcomes [1]. However, in advanced and recurrent settings, complete surgical cytoreduction reduces the risk of recurrence [2, 3]. Routine lymphadenectomy has been found to have neither a prognostic nor predictive role during surgery [4, 5]. Though adjuvant chemotherapy is often used in stage IC, poorly differentiated Sertoli Leydig tumours and in advanced disease, its usage has been found to have a limited role in preventing recurrence [6, 7]. Evidence from these retrospective studies has formed the basis of published guidelines for the direct evaluation and management of these rare tumours [8, 9], regarding treatment options and adjuvant treatment to optimize oncologic outcomes [10, 11].

It is, therefore, imperative to continue to report collated retrospective observations of these rare tumours. Hence, the aim of this exploratory study was to describe the clinicopathological profile and treatment outcome of SCOTs diagnosed and treated at a single institution, over 10 years.

## Methods

This retrospective cohort study was approved by the institutional review board (IRB NO: A13-25.10.2023). The patients nor the public were involved in the implementation and design of the study.

### Setting and patients

Patients who underwent surgery for SCOTs between 1st January 2011 to 31st December 2020 were identified from the department’s list of discharge summaries during this period using key words such as sex cord stromal neoplasms, ovarian neoplasm, complex ovarian mass and abnormal uterine bleeding. During this period, the department transitioned from being a general gynecologic unit, specializing in gynecologic cancers to a gynecologic oncology department involved in gynecologic oncology training on 1st October 2015.

Electronic outpatient records, operation records, discharge summaries and pathological records of patients operated for sex cord tumours were reviewed to obtain information about clinical presentation, prior treatment history, preoperative tumour marker profile, surgery done, histological diagnosis, adjuvant treatment and oncological outcome.

### Surgeries performed

Staging surgery comprised of inspection of the abdomen and pelvis, salpingo-oopherectomy, omentectomy besides lymph node biopsies or lymphadenectomy, peritoneal biopsies and hysterectomy. Patients desirous of preserving fertility underwent fertility-sparing surgery which was restricted to unilateral salpingo-ooopherectomy with preservation of contralateral adnexa and uterus. Interval surgery was defined as definitive surgery after the upfront initiation of chemotherapy. Completion surgery was offered to patients following primary inadvertent, oncologic incomplete surgery for ovarian mass such as ovarian cystectomy with a postoperative diagnosis of sex cord ovarian neoplasm. Secondary cytoreduction was defined as repeat surgery for recurrence of neoplasm after prior complete oncologic treatment. The cancer stage allocated to patients treated prior to 2014 was revised according to the International Federation of Gynaecology and Obstetrics (FIGO) 2014 staging of ovarian cancer.

### Statistical analysis

Overall survival was calculated from the date of initiation of cancer-specific treatment (upfront surgery or neoadjuvant chemotherapy) in this institution to the date of last follow up or death due to any cause in patients after excluding patients with benign ovarian tumours. Disease-specific survival was computed from the date of cancer-specific treatment initiation (upfront surgery or neoadjuvant chemotherapy) to the last follow up or death due to disease. Progression-free survival time was calculated from the date of cancer-specific treatment initiation (upfront surgery or neoadjuvant chemotherapy to the date of any recurrence or date of last visit, whichever occurred first. In patients who had completed surgery, survival was calculated from the date of cancer-specific treatment initiation. In patients who presented with recurrent tumours after having received primary treatment in a different institute, overall survival and progression-free survival in these patients were calculated from the time of treatment of recurrence in our institution, as patients did not often have details of the time of initiation of primary treatment. To maintain homogeneity, these patients were excluded during separate analysis of patients who primarily presented to our institution upfront, as they were at elevated risk of recurrence. FIGO stage III and IV at presentation, were analysed as advanced disease. Patients who had no gross macroscopic disease after surgery were considered to have had complete cytoreduction. Incomplete cytoreduction included optimal (< 1 cm) and suboptimal (>1 cm) clearance of disease. SPSS statistics version 21 (IBM, Armonk, New York, USA) was used for statistical analysis. Discrete and continuous variables were analyzed using chi square or Fischer test and paired -*t*-test/ ANOVA/ non parametric tests. Cox’s Regression model was utilised to calculate hazard ratios (HRs) and 95% confidence intervals (CIs). Progression-free survival and overall survival were plotted using the Kaplan–Meier method.

## Results

### Clinical presentation

**Figure S1.**
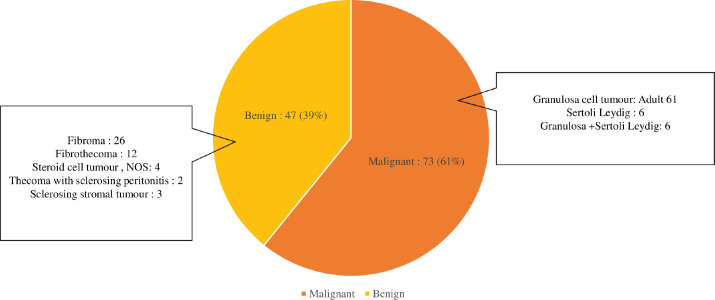
Description of ovarian sex cord pathology (*N* = 120).

**Figure S2.**
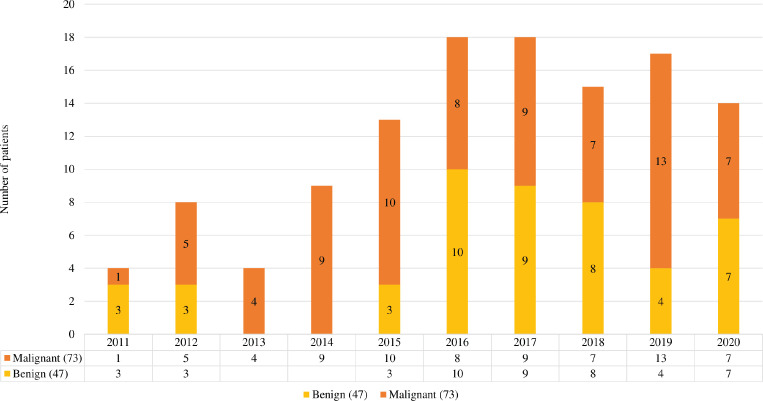
Sex cord tumours over 10 years.

Over the period of 10 years, there were 120 patients with SCOT who underwent surgery, of whom 57 (39%) were reported as benign in the final biopsy report (Supplementary Figure S1). There was a steady increase in the yearly number of SCOT over 10 years with a greater proportion of malignant SCOT (73,41%) tumours probably attributable to subspecialisation (Supplementary Figure S2). Granulosa cell tumour was the most reported malignant histology (61/73, 83.5%,). Malignant and benign SCOT were most common in the 4th decade (Table 1). Abnormal uterine bleeding was the most commonly reported presentation among patients with malignant ovarian tumours (28/120, 23.3%) of whom 12 of them presented with post-menopausal bleeding. However, hormonal manifestation, such as androgenisation, was similar in both groups. Anti-mullerian hormone (AMH) and Ca 125 were preoperatively assayed in 16 patients and 109 patients, respectively. Endometrial histological changes were seen in 29.8% of whom 2 had synchronous endometrial cancer in the uterine specimen, both of whom were post-menopausal with granulosa cell tumour. Malignant SCOT reported include granulosa cell tumours, Sertoli Leydig tumours and granulosa cell tumour with Sertoli Leydig components. Ovarian tumours with Sertoli Leydig components were common in younger aged adults (Table 2) and tumours with both granulosa and Sertoli Leydig histological components were significantly larger.

### Surgical evaluation

All patients who presented with SCOT primarily to the institution, with no prior treatment, underwent upfront surgery. During surgery, an intraoperative frozen section was utilised for 69 patients (57.5%), with a final biopsy discordancy rate of 11% among malignant ovarian tumours (4/36). Twenty-three (23/120, 19.1%) patients were offered fertility sparing surgery. Among patients with malignant SCOT, primary surgery was performed for 65 (65/73, 89.04%) of whom 12 (12/65,18.4%) underwent fertility preservation surgery (Table 3). Four (4/12) underwent oophorectomy with preservation of contralateral ovary after prior inadvertent cystectomy. Twelve patients (12/65, 18.4%) underwent completion surgery with removal of uterus and contralateral ovary, one of whom desired and underwent completion surgery after initiation of chemotherapy following fertility preservation surgery.

Eight with recurrence (8/73, 10.9%) at presentation to the institution after prior oncologic treatment, underwent cytoreduction of recurrence which included tertiary cytoreduction as the prior recurrence was previously operated.

Laparoscopic approach was performed in 4 among 56 patients with the apparent ovary-confined disease and lymphadenectomy in 26 patients with malignant SCOTs (35.6%). Three (3/25, 11.53%) patients had lymph node involvement. Added procedures such as diaphragm stripping/resection and small bowel resection were needed in 4 of the 16 (25%) with advanced/recurrent disease to obtain optimal cytoreduction (<1 cm) though complete cytoreduction could be achieved only in 9 (9/16, 56.25%). Major complications were rare with no 30-day mortality.

### Adjuvant treatment and oncologic outcome

Adjuvant chemotherapy was advised in 39 patients (53.4%) of whom 32 (43.8%) received the same (Table 3, Supplementary Table S1). Twenty-one (21/47, 44.6%) patients with early-stage disease received chemotherapy (FIGO IA-IC). Chemotherapy schedules employed were either paclitaxel or etoposide-based chemotherapy.

Over a median follow up period of 47 months (0–130 months), there were 11 recurrences (15.06%), (Supplementary Table S2). The 5-year recurrence rate of 9.58%. Four recurrences occurred later, after 5 years of initial treatment.

Four recurrences occurred in stage IA, 3 of whom had undergone fertility-sparing surgery, of whom, one opted for removal of the contralateral ovary and uterus after chemotherapy. Five recurrences occurred in advanced disease and two in those who had recurrence at first presentation and had undergone secondary and tertiary cytoreduction each. Seven had received adjuvant chemotherapy after the initial surgery. At the time of recurrence, five patients were eligible and underwent secondary cytoreduction. After diagnosis of recurrence, two did not follow up further, and six patients died of repeat recurrence/progressive disease, contributing to a disease-specific survival rate of 89.04% (6/73). There were four disease unrelated deaths among the patients who did not have recurrence.

### Risk factors for recurrence/death

**Figure S3.**
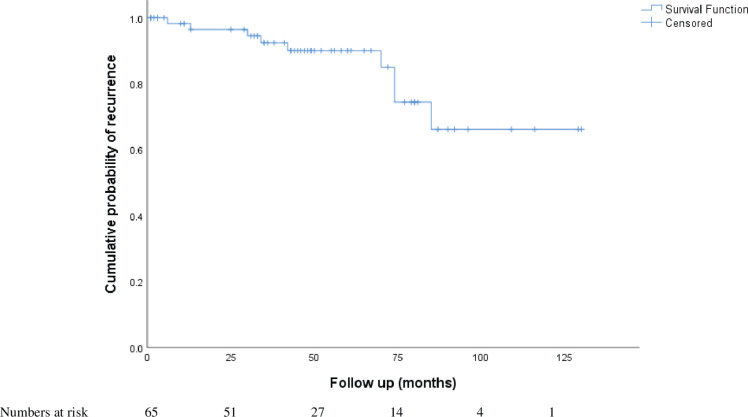
Kaplan-Meier curves of overall risk of recurrence in patients with upfront presentation of malignant sex cord ovarian tumours.

The overall mean recurrence-free period was 105.58 months (95% CI 92.89 to 118.27) and the mean overall survival period was 117.15 months (95% CI 107.56–126.74) (Supplementary Figure S3). On univariate analysis (Supplementary Table S3), younger age, advanced stage of disease, non granulosa histology and incomplete cytoreduction were found to be statistically associated with recurrence and death (4 deaths, unrelated to disease were excluded). However, on multivariate analysis (Supplementary Table S4), the advanced stage was a risk factor for recurrence (HR 21.57, 95% CI, 3.906–119.17, *p* = 0.00) and death (HR 73.925, 95% CI, 2.065–2,645.829, *p* = 0.018). Advanced age seemed to protect against death (HR 0.826, 95% CI 0.713–0.964, *p* = 0.015).

Among the 65 patients who had presented with upfront disease at presentation (Table 4), 9 (13.84%) developed recurrence over a median recurrence-free period of 46 months (1–65 months) with 4 disease-related deaths and 4 unrelated deaths. On univariate analysis, younger age (HR 0.934, 95% CI 0.881–0.991), incomplete cytoreduction (HR 58.391, 95% CI 5.042–674.854), advanced FIGO stage (HR 15.931, 3.74–67.89) and non granulosa histology were associated with risk of recurrence. On multivariate analysis, advanced FIGO stage (HR 20.099, 95% CI 3.75–107.711) and non granulosa histology (HR 31.35, 95% 2.801–350.897) remained significantly associated with recurrence. The performance of lymphadenectomy and the usage of adjuvant chemotherapy did not prevent recurrence.

In view of a few deaths (4 disease-related deaths), Cox regression was not performed to assess risk factors for death. Among the proportion of clinical characteristics (Supplementary Table S5) associated with disease-related deaths, the proportion of patients with age less than 40 years, incomplete cytoreduction, advanced FIGO stage and non granulosa histology is higher.

**Figure 1.**
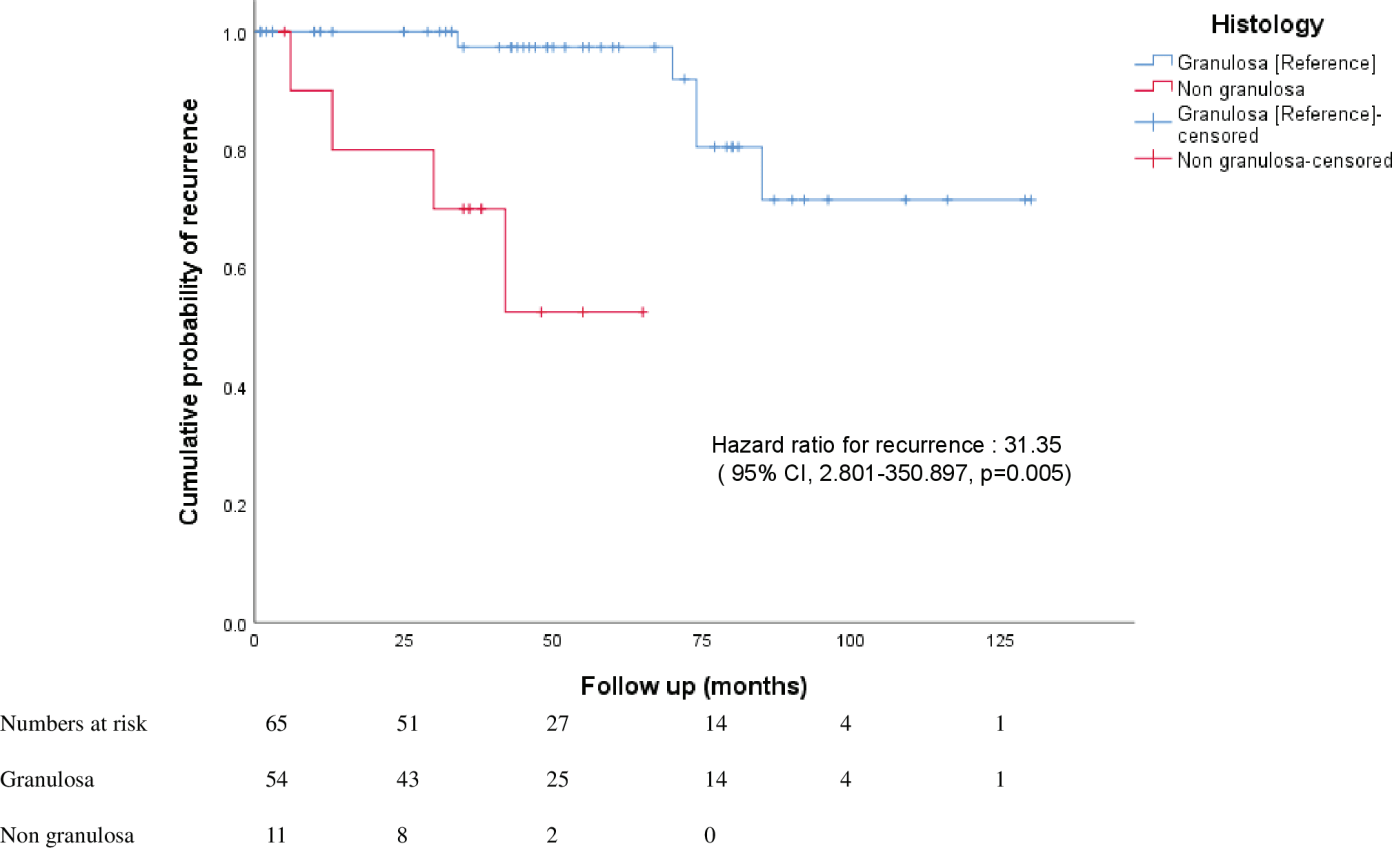
Kaplan-Meier curves of overall risk of recurrence in patients with upfront presentation of granulosa and non granulosa histology.

In this homogenous group, the overall mean recurrence-free period was 106.99 months (about 9 years) (95% CI, 93.810–120.178 months). Patients with granulosa cell histology had a mean recurrence-free period of 113.76 months (95% CI, 101.297–126.239). Patients with non granulosa histology (Sertoli Leydig, mixed granulosa-Sertoli) had a lower recurrence-free period (46.375 months, 95% CI 31.98–60.761, *p* = 0.000). On Cox regression analysis, non granulosa histology had a hazard risk of recurrence of 31.35 (95% 2.801–350.897) (Figure 1).

## Discussion

### Summary of main results

In this study, it was observed that as the department transitioned into subspecialisation, more patients with SCOT, with a greater proportion (73/120, 60.8%) of malignant tumours were treated. Granulosa cell tumour was the most common malignant histology. Though there was a change in practice over the years, a sizable number underwent lymphadenectomy. Adjuvant chemotherapy was utilised in 43.8%. Among those with an upfront presentation with sex cord malignant tumours, the recurrence rate was 13.84%. Patients with non granulosa histology and advanced disease were at elevated risk of recurrence. The performance of lymphadenectomy and utilisation of adjuvant chemotherapy did not prevent a recurrence.

### Discussion of results in the context of current literature

Sex cord tumours are a group of rare, heterogenous ovarian tumours which could be pure sex cord or pure stromal or a combination of sex cord and stromal tumours depending on the cell of origin [12]. Granulosa cell tumour is the commonest and is often seen in the 5th decade of life, which is later than the other SCOT.

### Clinical presentation

Granulosa ovarian tumours were the commonest in our cohort with late-onset presentation compared to other SCOT. Symptoms due to hormonal excess such as abnormal uterine bleeding could be due to hyperestrogenism as seen in Granulosa and Sertoli tumours whereas virilising manifestations, secondary to hyperandrogenism are seen in sclerosing stromal and Sertoli Leydig tumours [13, 14]. Abnormal uterine bleeding was mostly reported by our patients with malignant ovarian tumours and 2 (1.6%) patients had associated endometrial carcinoma on endometrial evaluation and endometrial hyperplasia (11.6%). These hormonal manifestations can be captured by measurement of serum AMH, Inhibin B and serum testosterone. AMH was measured in our patients after 2018 and Inhibin B measurement is still not available in our institution.

These tumours are typically unilateral and on average measure 10–15 cm in size [15] with early-stage presentation. The results of this study showed significantly larger sized tumour (median −22 cm) in tumours that had both Sertoli-Leydig and granulosa components.

### Surgical evaluation

Presumptive diagnosis of SCOT can be made before surgery based on classical endocrinological manifestations, tumour marker profile or based on the radiological description of heterogenous solid cystic masses or solid masses in young adults with hormonally inert tumours [16]. However, in young adults, the frozen section may help identify malignant SCOTs, differentiate them from benign SCOTs and metastatic carcinoma [17], which can help plan the extent of surgical staging and endometrial evaluation in case of fertility preservation surgery. Based on reports of low incidence of lymph nodal involvement in SCOT [18], routine lymphadenectomy is no longer recommended. However residual disease is a prognostic factor for recurrence and complete cytoreduction has been recommended in both advanced and recurrent SCOT [19].

Our results showed utilisation of frozen section in 2/3rds of patients; however, change in treatment plan could not be derived from this retrospective study. Around 16% of malignant ovarian tumours could undergo fertility preservation even after previous inadvertent cystectomy. The laparoscopic route was utilised in patients who required completion surgery or those in whom tumours could be removed without intrabdominal spillage. Though around 35% underwent routine lymphadenectomy, the department has changed its protocol to debulking of suspicious nodes only in sex cord and germ cell tumours over the recent years based on consistent recent evidence which has been incorporated into recommendations. Upfront advanced/recurrent disease was found in 15% of patients, 25% of whom required complex surgery. Though they could be optimally reduced, the complete cytoreduction rate among these patients was 56.25%. Residual disease which precluded complete cytoreduction was often due to small-volume mesenteric disease. The results of this study resonate with the absence of the benefit of routine lymphadenectomy and the significance of complete macroscopic clearance.

### Adjuvant treatment and oncologic outcome

Due to the indolent nature of the disease, SCOTs can present with late recurrences and are partially sensitive to chemotherapy. In high risk, early-stage disease (granulosa cell: stage I C or poorly differentiated Sertoli Leydi or with heterologous elements [9] adjuvant chemotherapy or observation has been recommended [20]. However, chemotherapy may benefit advanced disease [21, 22]. Bleomycin-etoposide-cisplatin (BEP) is the chemotherapy regimen often used. However, carboplatin-paclitaxel is used in older patients and to avoid bleomycin-related pulmonary toxicity [9]. There is no data directly comparing the efficacy of these two regimens and the choice of regimen is based on patient factors such as age, performance status and comorbid illnesses.

Our cohort showed a high utilization of adjuvant chemotherapy, even in early-stage disease for earlier quoted indications such as large tumour size and high mitotic rate [23, 24]. Recent evidence has shown capsular rupture or involvement to be a prognostic factor for relapse [25] but the usage of adjuvant chemotherapy has not been shown to decrease the risk [26]. In the absence of the benefit of adjuvant chemotherapy, the concerns regarding long-term complications of chemotherapy and its impact on quality of life, the department now has a multidisciplinary, individualized approach to patients with SCOTs to identify patients who would benefit from adjuvant chemotherapy.

Sex cord stromal tumours can recur later and hence require long-term surveillance. Due to poor response to chemotherapy, surgical excision of recurrence is advised. Chemotherapy in non operable cases will obtain objective response rates but anti hormonal therapy has been shown to have a higher disease control rate due to its ability to stabilize disease [27]. Chemotherapy or antihormonal therapy can be used after excision of recurrence to avoid 2nd recurrence. Anti-hormonal therapy options which include aromatase inhibitors (e.g., letrazole and anastrozole), progestins (megestrol acetate), selective estrogen receptor modulators (e.g., tamoxifen) and gonadotropin receptor antagonists may work by inhibition of proliferation of neoplastic cells directly through receptors on the tumour cells or indirectly by suppression of gonadotropins [28].

Poorly differentiated Sertoli Leydig tumours and advanced disease are negatively associated with high relapse rates but complete surgical staging even in recurrent cases is an important prognostic factor for survival [15]. Similarly, in our study, non granulosa histology and FIGO stages III and IV were at higher risk of recurrence, in a homogenous group of patients with primary sex cord malignant tumours.

The recent discovery of mutations in DICER1, STK11 and FOXL2 genes has improved our understanding in addition to the approach to sex cord stromal tumours and can serve as diagnostic and prognostic markers [15] and may assist in risk stratification in the future.

### Strengths and weaknesses

The strengths of this study include : 1 ) the study addresses the scarcity of research on SCOTs, offering insights from a single institution’s gynaecologic oncology department. 2) As the data spans a decade, it provides a longitudinal perspective, capturing changes in treatment guidelines over time and providing a reflection of real-world scenarios. 3) The uniqueness of the study lies in its exploration of cases treated by a gynaecologic oncology department that transitioned from a gynaecology department, potentially offering diverse perspectives.

The weaknesses of the study are like any retrospective study: 1) The study’s retrospective design limits the availability of documented indications for chemotherapy, introducing potential information gaps. 2) The diverse histology and low recurrence rate contribute to wide CIs, affecting the statistical significance of identified risk factors. 3) With a low incidence of deaths, the study faces limitations in calculating risk factors for disease-related deaths, potentially impacting comprehensive conclusions.

### Conclusion

Treatment of malignant sex cord stromal tumours should be referred to gynaecologic oncology specialists who can individualize surgery and adjuvant treatment to obtain maximal survival and ensure long-term quality of life. As prospective data on rare ovarian tumours are limited, collaborative rare tumour registries are recommended to develop new therapeutic strategies based on strong rationale.

## Conflicts of interest

The authors have no conflict of interest to declare.

## Funding

This research received no specific grant from any funding agency in the public, commercial or not-for-profit sectors’.

## Ethics statement

This study was approved by the institutional review board (IRB NO: A13-25.10.2023). In view of the retrospective nature of the study, patient consent was waived by the review board.

## Author contributions

MNS and VT conceived the study design. MNS, VT, AJ, RP, AS, DST, AT, RC and AP were involved in data collection, analysis, interpretation and conclusion. MNS and VT prepared the manuscript which was reviewed and approved by all the co-authors. All authors have agreed to be responsible for the published research.

## Figures and Tables

**Table 1.** General clinical characteristics (*N* = 120).

Clinical characteristics	Benign = 47	Malignant = 73	*p* value
Age at presentation (years, mean, SD)	47.7 (±14.8)	43.21 (±12.69)	0.07
BMI (mean, SD)	27.00 (± 7.18)	25.67 (±5.17)	0.943
Largest size of tumour (median, cm, IQR)	11.8 (8.15)	11.1 (7,16.6)	0.651
Symptoms Abnormal bleeding pattern (28.09%) Distension (20.66%) With thrombus (2.5%) Chronic pain (20.6%) Acute abdomen (9.09%) Androgenization (4.1% Pleural effusion (5.83%) Recurrence* Others	6 (5%) 10 (8.33%) 1 (0.83%) 16 (13.33%) 7 (5.83%) 2 (1.66%) 5 (4.16%) - -	28 (23.3%) 16 (13.33 %) 2 (1.66%) 9 (7.5%) 4 (3.33%) 3 (2.5%) 2 (1.66 %) 8 (10.9%) 1	0.002
Tumour markers Ca125 (unit/mL, median, IQR) AMH (ng, median, range)	34.3 (10.1,116.2) 0.41 (0.2–0.4)	18 (7.1,105) 7.37 (1.1, 23)	0.140
Endometrial histology Hyperplasia with/without atypia (11.6%) Endometrial carcinoma (1.66%) Hormonal effect (16.6%)	8 (6.66%) 0 5 (4.16%)	6 (5%) 2 (1.66%) 15(12.5%)	0.335

* The percentage was calculated with 73 malignant tumours as the denominator

SD, standard deviation; BMI, body mass index; IQR, Interquartile interval; AMH, antimullerian hormone

**Table 2.** Clinical characteristics of malignant SCOTs (*n* = 73).

Characteristics	Granulosa (61)	Sertoli Leydig (6)	Granulosa +Sertoli (6)	*p* value
Age (mean years, SD)	46.44 (±9.55)	23.17 (±6.21)	30.5 (±19.56)	0.00
BMI (mean,SD)	25.64 (±4.92)	26.53 (±6.39)	25.05 (±7.11)	0.145
Size of tumour (cm median, IQR)	10 (6.7,15)	10.45(5.75-16.25)	22 (16.27,25.5)	0.002
Stage IA (52.05%) IC (26.02%) II B (4.1%) III A (1.3%) III C (5.4%) Recurrence (10.9%)	32 16 2 1 3 7	3 1 - - 1 1	3 2 - - 1 -	

SD, standard deviation; BMI, body mass index; IQR, Interquartile interval

**Table 3.** Details of surgery and adjuvant treatment (malignant SCOTs, 73).

Surgical details	Granulosa (61)	Sertoli Leydig (6)	Granulosa + Sertoli (6)
**Usage of frozen section (49.3%)**	28	4	4
**Surgery performed** **Primary surgery (89.04%)** Fertility preservation (18.4%)* Completion surgery (18.4%)* **Surgery for recurrence (10.9%)** **No residual disease (93.1%)** **Optimal (<1 cm) (6.8%)**	54 *4* *12* 7 58 4	5 *4* - 1 5 1	6 *4* - - 1 -
**Laparoscopic approach (5.4%)**	3	1	-
**Lymphadenectomy (35.6%)**	21	2	3
**Extra pelvic surgeries** **Omentectomy/omental biopsy (90.4%)** **Diaphragm stripping/resection (2.7%)** **Small bowel resection/anastomosis (2.7%)**	56 - 2	5 1 -	5 1 -
Post operative complications ** ** Minor (16.4%)** ** Major (1.3%)**	11 1	1	
Adjuvant Treatment*** ** IA** ** IC** ** II B** ** IIIA** ** IIIC** **Recurrence**	27 (44.26%) 6 12 1 - 3 5	3 (50%) 2 - - - 1 -	2 (33.3%) - 1 - - 1

* The proportion of patients who during primary surgery underwent fertility preservation surgery/ completion surgery

** Clavien Dindo Classification: Minor: grade 1 and 2, Major: grade 3–5

*** Paclitaxel carboplatin: 18/32, Bleomycin etoposide cisplatin/etoposide cisplatin/carboplatin etoposide: 14/32

**Table 4.** Risk factors for recurrence following exclusion of patients who had presented with recurrence at presentation.

	Univariate analysis			Multivariate analysis		
Characteristics	HR	95% CI	*p* value	HR	95% CI	*p* value
**Age**	**0.934**	**0.881–0.991**	**0.024**	0.962	0.872–1.062	0.44
**Performance of lymphadenectomy**	1.219	0.318–4.677	0.773	3.539	0.32–39.191	0.303
**Incomplete cytoreduction**	**58.391**	**5.052–674.854**	**0.001**	0.23	0.001–39.83	0.57
**Advanced FIGO stage (III, IV)**	**15.937**	**3.741–67.898**	**0.000**	**45.282**	**2.140–957.995**	**0.014**
**Adjuvant chemotherapy usage**	0.367	0.091–1.471	0.157	0.502	0.086–2.927	0.444
**Non granulosa histology**	**23.018**	**2.547–208.046**	**0.005**	**21.993**	**0.552–875.754**	**0.1**

FIGO, International Federation of Gynecology and Obstetrics; HR, hazards ratio; CI, confidence intervals

Incomplete cytoreduction: any form of measurable macroscopic residual disease which includes optimal (< 1cm) and suboptimal (>1 cm) clearance of disease

Characteristics that are of statistical significance have been bolded.

## Supplementary

**Table S1.** Details of adjuvant treatment (expanded).

Histology	Adjuvant treatment			
	Adjuvant chemotherapy	Adjuvant hormones	Defaulted	No adjuvant treatment
**Granulosa cell (61)** ** IA** ** IC** ** II B** ** IIIA** ** IIIC** ** Recurrence**	27 (44.26%) 6 12 1 - 3 5	1 1	5 3 - 1 1 - -	28(45.9%) 23 4 - - - 1
**Sertoli Leydig (6)** ** IA** ** IC** ** IIIC** ** Recurrence**	3 (50%) 2 - 1 -		1 - - - 1	2 (33.3%) 1 1 - -
**Granulosa + Sertoli (6)** ** IA** ** IC** ** IIIC**	2 (33.3%) - 1 1		1 - 1 -	3 (50%) 3

Paclitaxel carboplatin: 18/32

Bleomycin etoposide cisplatin/etoposide cisplatin/carboplatin etoposide: 14/32

**Table S2.** Details of recurrence.

No	Year of primary treatment	Stage	Histology	Surgical treatment	Adjuvant chemotherapy	Recurrence free period (months)	Site of recurrence	Treatment of recurrence	Status at last follow up
1	2011	IA	Granulosa+ Sertoli	Fertility preservation	No	42	Peritoneum	Surgery & chemotherapy	Dead
2	2012	IA	Granulosa	Non fertility preservation	Paclitaxel carboplatin	34	Peritoneum	Surgery & chemotherapy	Defaulted
3	2013	IA	Granulosa +Sertoli	Fertility preservation	No	13	Peritoneum	Defaulted	Dead
4	2014	IIIC	Granulosa	Non fertility preservation	BEP	74	Peritoneum	Surgery followed by hormonal therapy	Alive
5	2015	IA	Granulosa	Fertility preservation	Carboplatin & etoposide followed by completion surgery	85	Pelvis	Surgery followed by hormonal therapy	Alive
6	2015	IIIC	Granulosa	Non fertility preservation	BEP	70	Peritoneum	Palliative intestinal diversion	Dead
7	2015	Recurrence	Granulosa	Tertiary cytoreduction	Etoposide Cisplatin	15	Peritoneum	Hormonal therapy	Dead
8	2016	Recurrence	Granulosa	Secondary cytoreduction	Hormonal therapy	9	Peritoneum	Hormonal therapy	Dead
9	2017	IIIC	Granulosa + Sertoli	Non fertility preservation	BEP	30	Peritoneum	Surgery and hormonal therapy	Alive
10	2017	IIIA	Granulosa	Non fertility preservation	Defaulted	74	Pelvis, liver	Hormonal therapy	Alive
11	2020	IIIC	Sertoli Leydig	Non fertility preservation	Paclitaxel carboplatin	6	Peritoneum	Hormonal therapy	Dead

BEP, bleomycin etoposide cisplatin

**Table S3.** Risk factor for recurrence (overall, *N* = 73).

	Univariate analysis			Multivariate analysis		
Characteristics	HR	95% CI	*p* value	HR	95% CI	*p* value
**Age**	**0.934**	**0.881–0.991**	**0.024**	0.962	0.872–1.062	0.44
**Performance of lymphadenectomy**	1.219	0.318–4.677	0.773	3.539	0.32–39.191	0.303
**Incomplete cytoreduction**	**58.391**	**5.052–674.854**	**0.001**	0.23	0.001–39.83	0.57
**Advanced FIGO stage (III, IV)**	**15.937**	**3.741–67.898**	**0.000**	**45.282**	**2.140–957.995**	**0.014**
**Adjuvant chemotherapy usage**	0.367	0.091–1.471	0.157	0.502	0.086–2.927	0.444
**Non granulosa histology**	**23.018**	**2.547–208.046**	**0.005**	**21.993**	**0.552–875.754**	**0.1**

FIGO, International Federation of Gynecology and Obstetrics; HR, hazards ratio; CI, confidence intervals

Incomplete cytoreduction: any form of measurable macroscopic residual disease which includes optimal (< 1cm) and suboptimal (>1 cm) clearance of disease

Characteristics that are of statistical significance have been bolded.

**Table S4.** Risk factors for death (overall, *N* = 69).

	Univariate analysis			Multivariate analysis		
Characteristics	HR	95% CI	*p* value	HR	95% CI	*p* value
**Age at presentation**	**0.926**	**0.867–0.988**	**0.034**	**0.826**	**0.713–0.964**	**0.015**
**Upfront presentation or at time of recurrence**	4.959	0.906–27.158	0.06			
**Performance of lymphadenectomy**	2.051	0.367–11.476	0.44			
**Incomplete cytoreduction**	**8.925**	**1.627–48.975**	**0.012**	0.95	0.101–8.972	0.964
**Advanced FIGO stage (III, IV, recurrence)**	**7.429**	**1.338–4.245**	**0.022**	**73.925**	**2.065–2,645.829**	**0.018**
**Adjuvant chemotherapy usage**	0.513	0.094–2.865	0.513			
**Non granulosa histology**	**7.627**	**1.481–39.27**	**0.015**	0.313	0.18–5.468	0.426

HR, Hazards ratio; CI, Confidence interval

Incomplete cytoreduction: any form of measurable macroscopic residual disease which includes optimal (< 1cm) and suboptimal (>1 cm) clearance of disease

Characteristics that are of statistical significance have been bolded.

**Table S5.** Clinical characteristics associated with disease related deaths after exclusion of patients who had recurrence at presentation.

Characteristics	Alive (*n* = 61)	Deaths (*n* = 4)	*p* value
**Age** **<40** **>40**	25 36	4	0.02
**Performance of lymphadenectomy** ** Yes** ** No**	21 40	2 2	0.398
**Cytoreduction** ** Complete** ** Incomplete**	59 2	3 1	0.004
**Figo stage** ** Early (stage I, II)** ** Advanced (III, IV)**	57 4	2 2	0.004
**Adjuvant chemotherapy usage** ** Yes**	25 36	2 2	0.398
**Histology** ** Granulosa** ** Non granulosa histology**	53 8	1 3	0.001

FIGO, International Federation of Gynecology and Obstetrics

Incomplete cytoreduction: any form of measurable macroscopic residual disease which includes optimal (< 1cm) and suboptimal (>1 cm) clearance of disease
